# T Cell Receptor and Cytokine Signaling Can Function at Different Stages to Establish and Maintain Transcriptional Memory and Enable T Helper Cell Differentiation

**DOI:** 10.3389/fimmu.2017.00204

**Published:** 2017-03-03

**Authors:** Sarah L. Bevington, Pierre Cauchy, David R. Withers, Peter J. L. Lane, Peter N. Cockerill

**Affiliations:** ^1^Institute of Cancer and Genomic Sciences, Institute of Biomedical Research, University of Birmingham, Birmingham, UK; ^2^Institute of Immunology and Immunotherapy, Institute of Biomedical Research, University of Birmingham, Birmingham, UK

**Keywords:** chromatin, enhancer, epigenetics, gene regulation, immunological memory, T cells, transcription

## Abstract

Experienced T cells exhibit immunological memory *via* a rapid recall response, responding to restimulation much faster than naïve T cells. The formation of immunological memory starts during an initial slow response, when naïve T cells become transformed to proliferating T blast cells, and inducible immune response genes are reprogrammed as active chromatin domains. We demonstrated that these active domains are supported by thousands of priming elements which cooperate with inducible transcriptional enhancers to enable efficient responses to stimuli. At the conclusion of this response, a small proportion of these cells return to the quiescent state as long-term memory T cells. We proposed that priming elements can be established in a hit-and-run process dependent on the inducible factor AP-1, but then maintained by the constitutive factors RUNX1 and ETS-1. This priming mechanism may also function to render genes receptive to additional differentiation-inducing factors such as GATA3 and TBX21 that are encountered under polarizing conditions. The proliferation of recently activated T cells and the maintenance of immunological memory in quiescent memory T cells are also dependent on various cytokine signaling pathways upstream of AP-1. We suggest that immunological memory is established by T cell receptor signaling, but maintained by cytokine signaling.

## Introduction

The adaptive immune system relies on both specific antigen (Ag) recognition and on the ability of lymphocytes to maintain a memory of previous encounters with Ags. These two features represent the essential basis of acquired immunity. The capacity of the immune system to recognize billions of epitopes is established *via* the complex process of Ag receptor gene recombination that produces countless combinations of specificities for foreign Ags. The ability of T cells to respond faster and more efficiently to weaker stimuli is supported by memory T cells which exhibit what is referred to as a rapid recall response (1–10). What was until recently not so well defined are the molecular mechanisms that actually allow memory T cells to respond much more rapidly to re-exposure to the same Ags. Recent studies have now shown that the acquisition of T cell-dependent memory is supported by the epigenetic reprogramming of the genome *via* T cell receptor (TCR) signaling. Activation of the TCR triggers a hit-and-run mechanism whereby a single cycle of activation leads to the acquisition of thousands of stably maintained active chromatin regions which include many of the inducible immune response genes that deliver effective immune responses (10). Active chromatin priming is now known to be one of several parallel mechanisms employed by activated T cells and memory T cells to enable the rapid expression of immune response factors. It is also established that activated T cells induce cytokine or chemokine production by virtue of enhanced TCR signaling (11, 12), loss of repressive chromatin modifications (13–15), increased mRNA stability (16), and more efficient translation of cytokine mRNAs (17). However, some of these mechanisms are only relevant for a subset of immune response genes (18), whereas active chromatin modifications represent a more universal mechanism of maintaining immunological memory throughout the T cell compartment (10). In this review, we will focus on just the role of active chromatin priming in T cells and present some new analyses of previously published data to illustrate the potential of TCR-inducible chromatin priming in underpinning the subsequent stages of T cell differentiation.

## T Cell Activation and Differentiation

**Figure 1:**
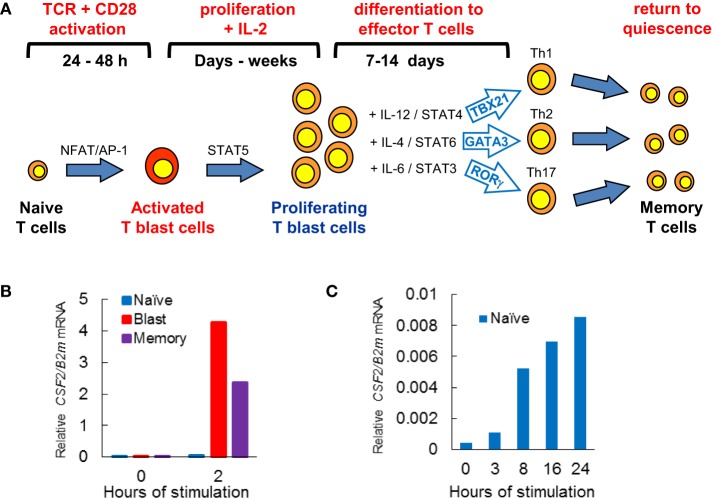
**T cell activation pathways linked to immunological priming**. **(A)** Naïve T cells are transformed by T cell receptor (TCR) signaling, leading to cytokine-dependent proliferation and differentiation, before reverting to quiescent memory T cells. **(B,C)** Quantitative PCR analyses of CSF2 mRNA expression relative to B2M mRNA expression in T cell subsets derived from CSF2 transgenic mice (23). Previously activated CD4 blast cells and memory T cells rapidly induce CSF2 mRNA when TCR signaling pathways are activated by PMA and calcium ionophore **(B)** (10). **(C)** Naïve CD4 T cells are very slow to respond to activation of TCR signaling by concanavalin A (10).

Mature T cells exit the thymus with all the genetic components needed to recognize Ags. However, what these naïve T cells lack is the ability to respond rapidly to their first encounter with the Ags recognized by their specific TCRs. During a productive immune response, when naïve T cells are first activated, they require correct Ag presentation over an extended period of time (~1 to 2 days) as they undergo the complex process of blast cell transformation. During this process they convert from small quiescent cells to larger highly proliferative cells (Figure 1A). Depending upon the nature of the Ag and the cytokine milieu in the environment where they reside, recently activated T cells can undergo further differentiation steps giving rise to different sub-types of effector T cells, expressing different combinations of immune response genes (19–22). For example, under the influence of IL-12 and STAT4, naïve CD4 cells tend to differentiate into type 1 helper (Th1) cells which can express inducible genes such as *IFNG* and *GZMB* which are activated *via* cooperation between the transcription factor (TF) TBX21 (T-Bet) and TCR-inducible TFs (Figure 1A). Conversely, IL-4 and STAT6 signaling in CD4 T cells triggers differentiation into type 2 helper (Th2) cells expressing TCR-inducible genes such as *IL4* which are activated by the TF GATA3. Recently activated T blast cells and differentiated T cells remain tightly regulated and rely on ongoing activation of TCR signaling to express inducible immune response genes (18).

## The Basis of the Rapid Recall Immunological Response in Memory T Cells

Once an immune response has been resolved, the vast majority of activated T cells die. However, a small proportion of cells return to the quiescent state as Ag-specific memory T cells. Unlike naïve T cells, memory T cells are primed and ready to respond to any new encounter with the same Ag. Indeed, a defining feature that memory T cells share with recently activated T blast cells is that they are capable of responding to TCR signaling to activate hundreds of immune response genes within 1–2 h (Figure 1B), whereas naïve T cells typically take about 24 h to mount a full response (Figure 1C) (10). The process of T blast cell transformation, which is required to make the rapid recall response possible, involves extensive chromatin remodeling, whereby the Brg1 SWI/SNF family chromatin remodeling complex mediates epigenetic reprogramming of the genome to prime inducible loci for transcriptional reactivation (24). This hit-and-run mechanism, triggered by TCR signaling, establishes thousands of open chromatin regions embedded within extensive active chromatin domains (10, 18). Once formed, these primed regions are stably maintained by mechanisms independent of TCR signaling. Indeed, hit-and-run mechanisms of chromatin priming are widely used in eukaryotes from plants to animals to maintain transcriptional memory (25–29). An example of this is found in previously activated macrophages which maintain regions marked by histone methylation in the vicinity of inducible enhancers (30).

## Defining the Epigenetic Landscape in T Cells

**Figure 2:**
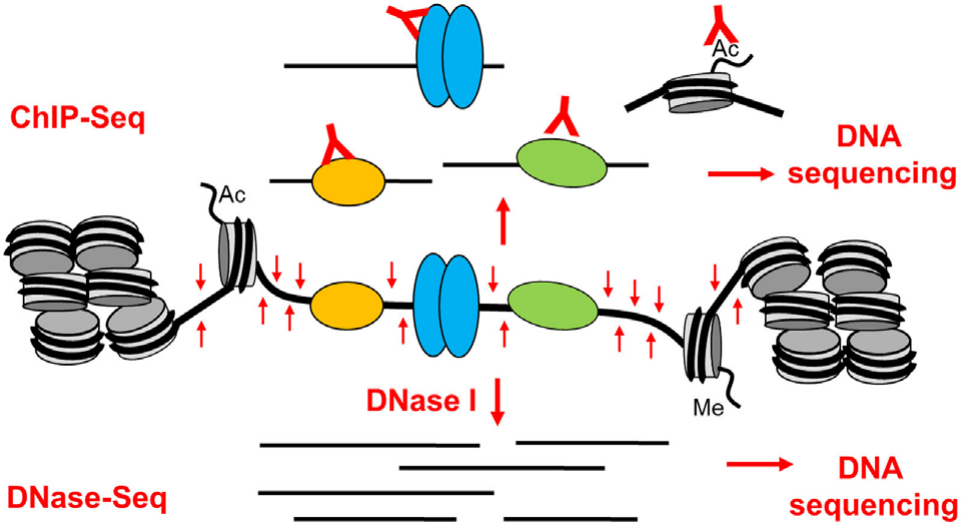
**The basis of epigenetic profiling of DNase I hypersensitive sites (DHSs) by DNase-Seq and ChIP-Seq**. Active gene regulatory elements that exist as open chromatin regions can be identified in a genome-wide manner by DNA sequencing of either (1) DNA fragments immunoprecipitated by specific antibodies from sonicated cross-linked chromatin (ChIP-Seq) or (2) small DNA fragments released from DHSs by brief digestion of chromatin with DNase I (DNase-Seq).

Studies of chromatin architecture and analyses of TF binding within chromatin have always played a major role in studies of gene regulation and genome function (31–37). These studies have collectively shown that active gene promoters and transcriptional enhancers typically exist as accessible nucleosome-free regions known as DNase I hypersensitive sites (DHSs) (31, 37). When active, these open regions are also normally flanked by nucleosomes carrying activating chromatin modifications such as acetylation and methylation of specific lysines in the histone tails (36–39). Active enhancer elements are typically associated with mono- and di-methylation of lysine 4 and acetylation of lysine 27 in the histone H3 tail (H3K4me1/2 and H3K27ac) (37, 39). Analyses of epigenetic mechanisms are now routinely done globally, and high-throughput sequencing technologies make it straightforward to perform complex integrated chromatin and gene regulation analyses on a genome-wide basis. By sequencing small DNA fragments released from DHSs by DNase I (DNase-Seq) or transposase (ATAC-Seq) (40), and DNA fragments cross-linked to immunoprecipitated TFs or modified histones (ChIP-Seq) (Figure 2) it is possible to define complex gene regulation networks (41).

Previous studies in a multitude of model systems have reported that gene loci can be activated in a step-wise manner whereby DHSs are acquired in a defined sequence as cells follow a specific differentiation pathway. Interestingly, this leads to the acquisition of some DHSs which are associated with genes that exist in a transcriptionally competent but inactive state. Examples of DHSs that are acquired through differentiation are observed when CD4 T cells are polarized to alternate fates as Th1 and Th2 cells. Hence, Th1-specific DHSs are associated with binding of TBX21 at the Th1 gene *IFNG*, and Th2-specific DHSs associated with GATA3 binding at the Th2 gene *IL4* (42). The DHSs in these cells are also associated with STAT proteins in a cell type-specific manner. STAT4 and STAT6 binding is observed at the DHSs in Th1 and Th2 cells, respectively (43, 44). However, in addition to differentiation-specific DHSs, CD4 T cells acquire many DHSs in genes such as *IL4* in recently activated T cells prior to terminal differentiation (10, 42). These newly acquired DHSs represent the early stages of acquiring transcriptional competency prior to the activation of active transcription (18, 23, 45).

It is also well established that extensive histone methylation and acetylation exists at genes poised for reactivation in T cells (8, 9, 46–48). Some genes in T cells exist in a poised state maintained by H3K4 di- or tri-methylation which is established by trithorax family SET domain proteins (28, 49, 50). In memory T cells, a proportion of immune response genes have their promoters marked by H3K4me3, whereas these genes are associated with repressive H3K27me3 modifications introduced by polycomb group complexes in naïve T cells (13, 46, 51–56). Both CD4 and CD8 memory T cells retain extensive histone acetylation and low levels of DNA methylation at immune response genes (48, 57–63). This is in contrast to the higher levels of DNA methylation observed in naïve T cells (14, 15, 42, 58, 61, 64). These epigenetic differences between naïve and memory T cells suggest that in memory T cells, immune response genes are maintained in a primed conformation. However, it was not always clear how these various chromatin states were targeted to specific loci at the various stages or how they were maintained. With the exception of the loss of DNA methylation, which represents a logical target for hit-and-run mechanisms of locus activation, active chromatin states typically rely on the continued presence of specific activating TFs. In the case of memory T cells, the specific factors responsible for maintaining chromatin priming were until recently poorly defined.

## Global Analysis of Chromatin Priming in Previously Activated T Cells

**Figure 3:**
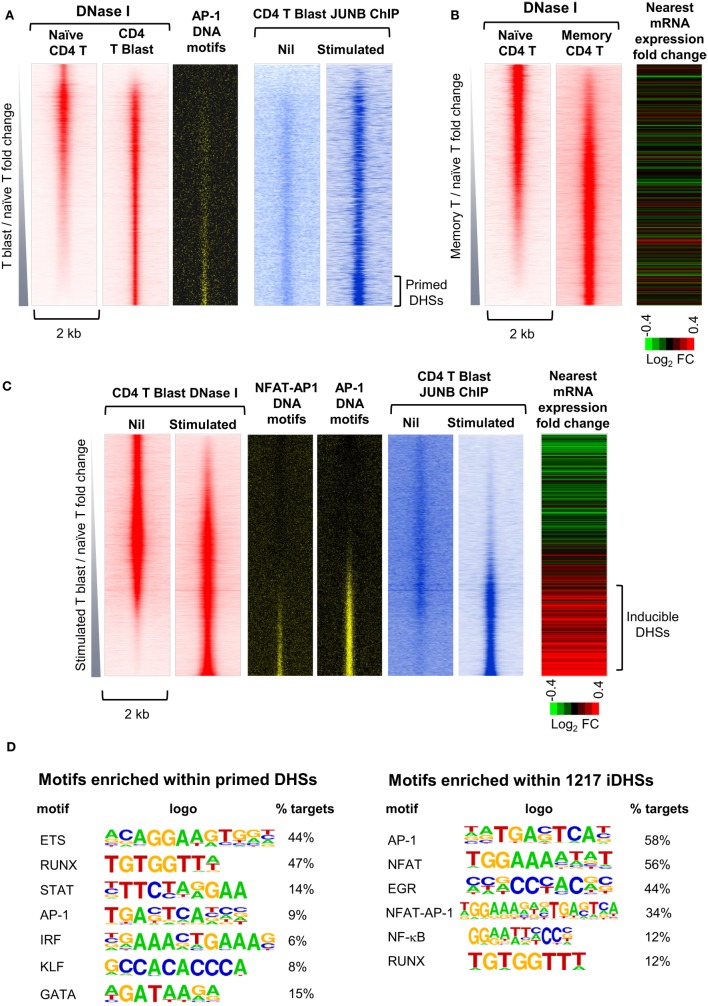
**Identification of distinct subsets of primed DHSs (pDHSs) and inducible DHSs (iDHSs) in mouse T cells by DNase-Seq and ChIP-Seq**. **(A)** Genome-wide profiling of pDHSs enriched in CD4+ T blast cells relative to naïve T cells (10). Profiles are shown for the ~17,000 strongest DNase I hypersensitive sites (DHSs) present in naïve and/or T blast cells, centered on a 2 kb window and ranked in order of increasing intensity in T blast cells. Shown alongside are the positions of predicted binding motifs for AP-1, and data from parallel JUNB ChIP-Seq analyses of AP-1 binding for the same DHSs in T blast cells before and after stimulation with PMA and calcium ionophore. **(B)** Genome-wide profiling of pDHSs enriched in CD4+ memory T cells relative to naïve T cells (10). Profiles are shown for all DHSs centered on a 2 kb window and ranked in order of increasing intensity in memory T cells. Shown alongside is a heat map depicting the fold change in mRNA expression of the genes nearest to these DHSs following stimulation with PMA and calcium ionophore. **(C)** Genome-wide profiling of iDHSs enriched in stimulated CD4+ T blast cells relative to unstimulated cells (10). Profiles are shown for all DHSs centered on a 2-kb window and ranked in order of increasing intensity in stimulated T blast cells. Shown alongside are the positions of predicted AP-1 and composite NFAT/AP-1 binding motifs, and data from parallel JUNB ChIP-Seq analyses of AP-1 binding for the same DHSs in T blast cells before and after stimulation with PMA and calcium ionophore, plus a heat map depicting the fold change in mRNA expression of the genes nearest to these DHSs following stimulation. **(D)** HOMER analysis of transcription factor-binding motifs enriched in either pDHSs or iDHSs (10).

To gain a greater understanding of epigenetic priming in T cells, we recently embarked on an in depth study of the chromatin modifications are first acquired occur during T blast cell transformation and are then maintained in differentiated T cells and memory T cells (10). By identifying all of the DHSs that are present in recently activated murine CD4 T blast cells and CD4 memory T cells, but not in naïve CD4 T cells, we identified ~3,000 newly acquired and stably maintained primed DHSs (pDHSs) that could potentially account for immunological memory in T cells (Figures 3A,B) (10). These DHSs fulfilled some of the essential features needed for a priming mechanism, because they supported the maintenance of domains of active chromatin modified by H3K4me2 and H3K27ac without having any significant impact on steady-state levels of transcription (10, 18) (Figure 3B). Consistent with this, transcriptional memory has been associated with a retention of H3K4me2 in other systems (27, 65).

Significantly, the primed active domains seen in T cells often encompassed inducible DHSs (iDHSs) which are known to function in the TCR-dependent activation of immune response genes. The inducible enhancers associated with these pDHSs included those which regulate genes encoding the inducible cytokines IL-3, GM-CSF (CSF2), IL-13, and IL-4 (10, 23, 66–68). These enhancers are included in a population of several thousand highly iDHSs, which are closely associated with inducible genes in recently activated T blast cells (Figure 3C). The inducible nature of these loci is depicted here as a heat map showing the relative change in mRNA for the nearest gene to each iDHS following stimulation.

Somewhat paradoxically, pDHSs and iDHSs generally recruit the same classes of TFs, but in different proportions (Figure 3D). However, this makes perfect sense given that there is no substantial change in the TF repertoire immediately following T blast cell transformation, but only later in the differentiation process (Figure 1A). The iDHSs are highly enriched with binding sites for the TCR-inducible TFs NFAT and AP-1 (Figures 3C,D), as well as motifs for EGR and NF-kB family TFs which can be induced *via* multiple signaling pathways (10). More than half of these NFAT and AP-1 motifs exist as composite NFAT/AP-1 motifs, which are known to support cooperative binding of NFAT and AP-1, as previously described for the IL-2 promoter and the GM-CSF enhancer (69, 70). When aligned with the iDHSs, the AP-1 and AP-1/NFAT motifs are heavily concentrated in the inducible population, and they recruit the AP-1 protein JUNB upon stimulation (Figure 3C). By contrast, the pDHSs encompass far fewer AP-1 motifs (Figure 3D), but nevertheless they still contain significantly more AP-1 sites than the other constitutive DHSs shared with naïve T cells (Figure 3A). Importantly, the AP-1 motifs in pDHSs can recruit the AP-1 TF JUNB, but only in response to stimulation (Figure 3A). The pDHSs are principally defined by the high density of RUNX1 and ETS-1 motifs, whereas they have a much lower density of inducible binding sites. Although they lack NFAT sites, subsets of these elements retain the ability to respond to various signaling pathways as they include motifs for STAT, AP-1, and IRF family TFs, and for the STAT6-inducible TF GATA3 (Figure 3D). The iDHSs also respond by recruiting constitutive TFs such as RUNX, but unlike the pDHSs, they lose binding of RUNX1 once the inducible factors have gone.

## Studies of the Th2 Cytokine Gene Locus as a Model for Chromatin Priming

**Figure 4:**
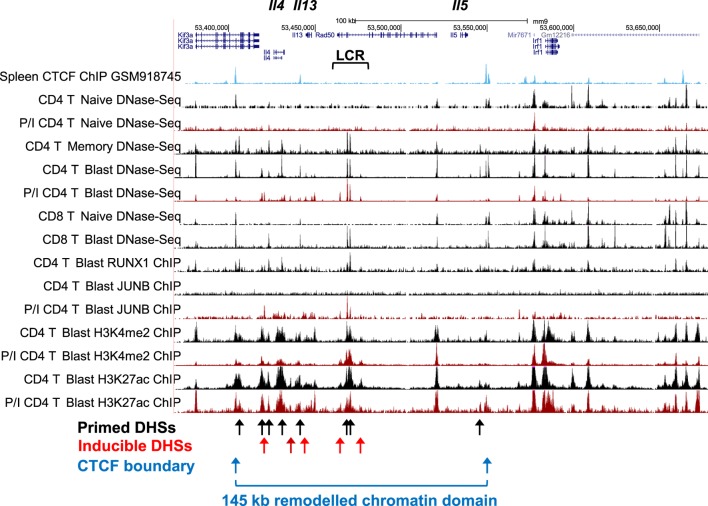
**Profiling of epigenetic modifications in the mouse type 2 helper cytokine gene cluster by DHS-Seq and ChIP-Seq**. Profiles are based on published data from naïve, blast, and memory T cells (10) and include published data for CTCF ChIP-Seq from splenocytes (74).

The Th2 cytokine gene cluster encompassing *Il4, Il13*, and *Il5* (Figure 4) represents an ideal model for depicting the key features of the pDHSs and iDHSs at the different stages of T cell activation, expansion, and differentiation illustrated above in Figure 1A. These genes reside within a 145 kb chromatin domain demarcated by two prominent binding sites for the insulator factor CTCF which may represent chromatin boundaries, as previously shown for a cluster of CTCF sites in the *IL3/CSF2* locus (71). The Th2 cytokine gene locus is controlled by a single locus control region (LCR) which resides within the *Rad50* gene between *Il13* and *Il5* (72, 73). Significantly, with the exception of the *Rad50* promoter, this entire 145 kb chromatin domain exists in an inaccessible and unmodified state in naïve T cells, as measured by DNase-Seq and H3K4me2 and H3K27ac ChIP-Seq (Figure 4). By contrast, the *Irf1* chromatin domain downstream of the 3′ CTCF site is packed with constitutive DHSs in both naïve T cells and T blast cells.

After a cycle of TCR activation and blast cell transformation, actively dividing T blast cells cultured in IL-2 have acquired and stably maintain many additional DHSs within the Th2 cytokine gene locus. These pDHSs persist in non-dividing CD4 memory T cells and are also observed in CD8 T blast cells (Figure 4) and Th2 T cells (75). Because the vast majority of the ~3,000 CD4 T cell pDHSs were also detected in CD8 T blast cells, it is likely that the mechanism of chromatin priming seen here is a universal feature of T lineage cells (10). The significance of the presence of the pDHSs becomes obvious once it is recognized that the pDHSs are embedded within extensive active chromatin domains carrying the activating H3K4me2 and H3K27ac modifications (Figure 4). Furthermore, these active domains encompass several iDHSs which are known to function as transcriptional enhancers (68). The inference here is that the pDHSs are functioning to render the adjacent enhancers more accessible, and therefore more responsive to TCR signaling. This is exemplified by the rapid recruitment of the AP-1 family TF JUNB in stimulated T-blast cells to iDHSs (Figures 3C and 4). By contrast, these enhancers fail to form iDHSs in stimulated naïve CD4 T cells and thymocytes (Figure 4), even though the AP-1 and NFAT mRNAs required for enhancer activation are efficiently expressed by activated naïve T cells (10, 23, 45).

## Studies of the Human *IL3/CSF2* Locus as a Model for Chromatin Priming

Alongside the *IL2* and Th2 cytokine gene loci, the human *IL3/CSF2* locus is one of the best studied models of inducible cytokine gene regulation in T cells. The use of transgenic mouse models in parallel with human and mouse cells has made it possible to investigate the developmental regulation, the inducible regulation, and the conservation of regulatory elements at this locus (10, 23, 45, 71, 76–79). Analyses comparing thymocytes, spleen T cells, and T blast cells were central to the original identification of DNA priming elements associated with locus priming and transcriptional competency in T cells (23). Similar to the Th2 cytokine locus, the human *IL3/CSF2* locus exists as a tract of greater than 100 kb of unmodified inactive chromatin in naïve T cells, and it acquires several pDHSs and active chromatin domains during T blast cell transformation. Following blast cell transformation, several iDHSs which function as promoters and enhancers can then be induced within the region (10). Consistent with what we observed at the human locus, the mouse *Il3/Csf2* locus showed conservation of most of the pDHSs and iDHSs, and in this case the inactive chromatin domain spanned at least 220 kb in naïve mouse CD4 T cells (10). Parallel functional analyses of pDHSs in the *IL3/CSF2* locus revealed that, as a general rule, pDHSs, unlike iDHSs, tend not to function directly as transcriptional enhancers but as DNA elements that may be required to maintain zones of activating histone methylation and acetylation (23, 45). Along with similar observations made by others, the body of evidence now indicates that the mere presence of active chromatin modifications at DHSs is not in itself sufficient to predict enhancer function (80), even though this remains a popular assumption.

The above studies also suggested that, once established in response to TCR activation, pDHSs were stably maintained in actively dividing T cells in the presence of IL-2, and were temporally stable in circulating human T cells for what must be assumed to be decades (10, 23). What remains to be seen is whether ETS and RUNX TFs remain bound to chromatin during mitosis, as seen for GATA1 which functions as a mitotic bookmarking factor in erythroid lineage cells (81).

We confirmed that pDHSs can indeed cooperate functionally with inducible enhancers by deleting the human *IL3* −34 kb pDHS from its natural context in Jurkat T cells. This deletion led to a marked decrease in chromatin accessibility at the nearby −37 kb IL3 enhancer, in parallel with a change in kinetics of *IL3* mRNA induction from a pattern resembling that seen in memory T cells (as in Figure 1B) to a pattern resembling that seen in naïve T cells (as in Figure 1C) (10). From these studies, it was apparent that pDHSs did indeed function locally at the level of chromatin accessibility, and globally there was a trend for pDHSs to reside within 25 kb of iDHSs at inducible loci (10). The most highly inducible genes were also found to be the loci where pDHSs and iDHSs were the closest, independently of their distance from the inducible promoters that they controlled.

## A Hit-and-Run Model of Immunological Memory in T Cells

**Figure 5:**
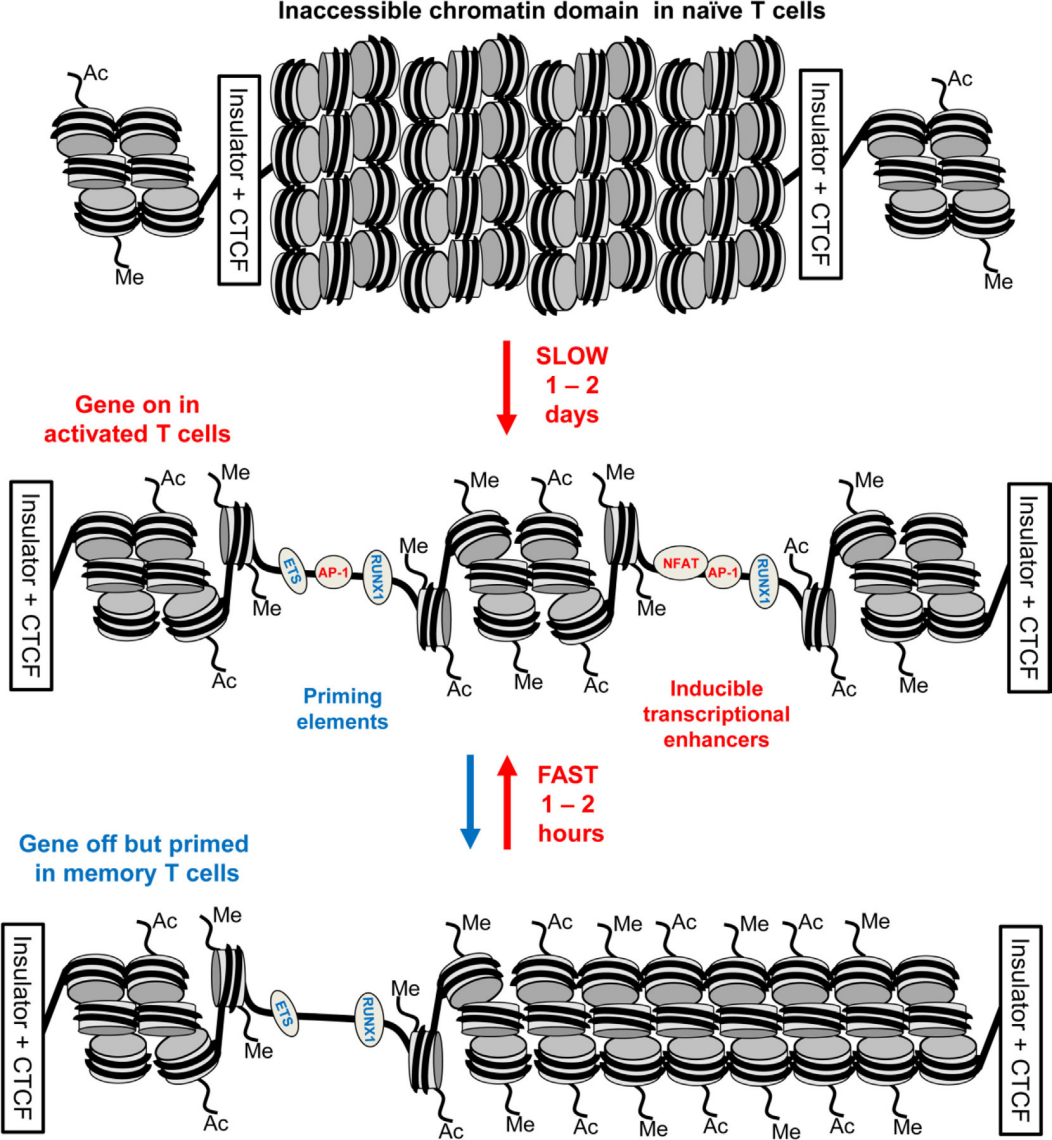
**Chromatin priming in T cells supports a rapid recall response**. This model represents a closed chromatin domain, resembling that seen in Figure 4, which undergoes epigenetic priming in T blast cells to maintain an accessible active chromatin structure. The primed domain, but not the closed domain, allows for rapid recruitment of inducible factors. The primed DHSs are formed *via* a hit-and-run mechanism whereby inducible transcription factors (TFs) are required for the initial induction but not the stable maintenance of these DNase I hypersensitive sites (DHSs). By contrast, the inducible DHSs found at inducible enhancers form transient DHSs that are unstable in the absence of inducible TFs.

Taken together, these observations support a hit-and-run model for the initial establishment of epigenetic priming during T blast cell transformation which is predicted to alter chromatin accessibility (10) (Figure 5). Within the nucleus, most chromatin is thought to be compacted into condensed structures ~110 to 170 nm in diameter (82), which is roughly fivefold greater than the classical 30 nm diameter solenoidal structure depicted at the bottom of Figure 5. Many immune response genes lie within large tracts of unmodified and inaccessible chromatin in thymocytes and naïve T cells, and this is likely to present a formidable barrier to inducible TF binding. Therefore, although ETS-1 and RUNX1 are already bound to the constitutive DHSs shared by naïve T cells and T blast cells (10), we believe that the chromatin harboring pDHSs is simply inaccessible to these TFs in naïve T cells. It is only after an extended period of TCR signaling and Brg1-dependent chromatin remodeling that the combined actions of AP-1, ETS-1, and RUNX1 are sufficient to open up pDHSs and modify the surrounding chromatin. This process of *de novo* DHS creation will also be considerably enhanced by the actions of NFAT and AP-1 at the adjacent enhancers during blast cell transformation. These factors are thought to be powerful drivers of chromatin opening and remodeling (24, 78). Once activated, the pDHSs are presumably maintained due to the high density of constitutive TF-binding sites, and consequently the surrounding chromatin is continuously remodeled and rendered more accessible. This more active chromatin structure may enable the primed immune response genes to more rapidly re-engage AP-1 and other inducible factors at both pDHSs and iDHSs when the cells are re-challenged. Furthermore, the presence of extensive histone acetylation and methylation is likely to make these chromatin domains even more accessible and the nucleosomes more mobile. This view is supported by the fact that the iDHS at the GM-CSF enhancer is strongly induced in just 20 min in T blast cells (78), but this and many other iDHSs remain undetectable even after 4 h of stimulation in naïve T cells and thymocytes (10, 23, 45). The binding of RUNX1 to the GM-CSF enhancer (83), and other iDHSs (10), is also controlled at the level of chromatin accessibility, because RUNX1 binding is only observed after the DHS has been induced. Because pDHSs exist in both CD4 and CD8 T blast cells, the hit-and-run epigenetic priming model presented in Figure 5, and exemplified in Figure 4, is likely to be a universal component of immunological memory in T lineage cells. Another important feature of this model of chromatin priming is that pDHSs allow genes to remain in a poised state with essentially no change in steady-state levels of transcription of primed loci in primed cells as compared to naïve T cells (10, 18). An analysis of the *IL3/CSF2* locus also found that pDHSs do not recruit RNA Pol II or generate non-coding transcripts in the absence of stimulation (23).

## The Role of Chromatin Priming in Supporting T Cell Differentiation

**Figure 6:**
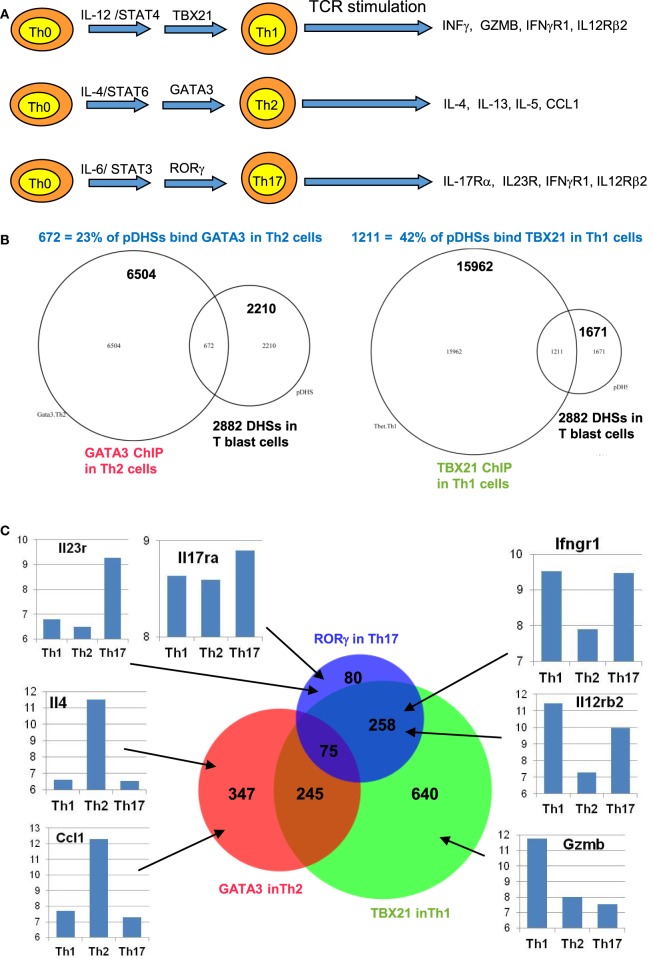
**Chromatin priming in undifferentiated CD4 T cells creates open chromatin at regions encompassing binding sites for differentiation-inducing transcription factors**. **(A)** Schematic of differentiation pathways leading to type 1 helper (Th1), type 2 helper (Th2), and Th17 cells. **(B)** Venn diagrams showing overlaps of previously defined ChIP-Seq peaks for GATA3 in Th2 cells (84) (left) and for TBX21 (T-Bet) in Th1 cells (85) (right) with the 2,882 primed DHSs (pDHSs) previously defined in CD4 T cells (10) as depicted in Figure 3. **(C)** Venn diagram depicting the overlap of Th2 GATA3, Th1 TBX21, and Th17 RORγ (86) ChIP-Seq peaks intersecting with the 2,882 pDHSs, with examples of corresponding gene expression patterns previously defined in Th1 (87), Th2 (88), and Th17 (87) cells for representative genes that are associated with pDHSs.

**Figure 7:**
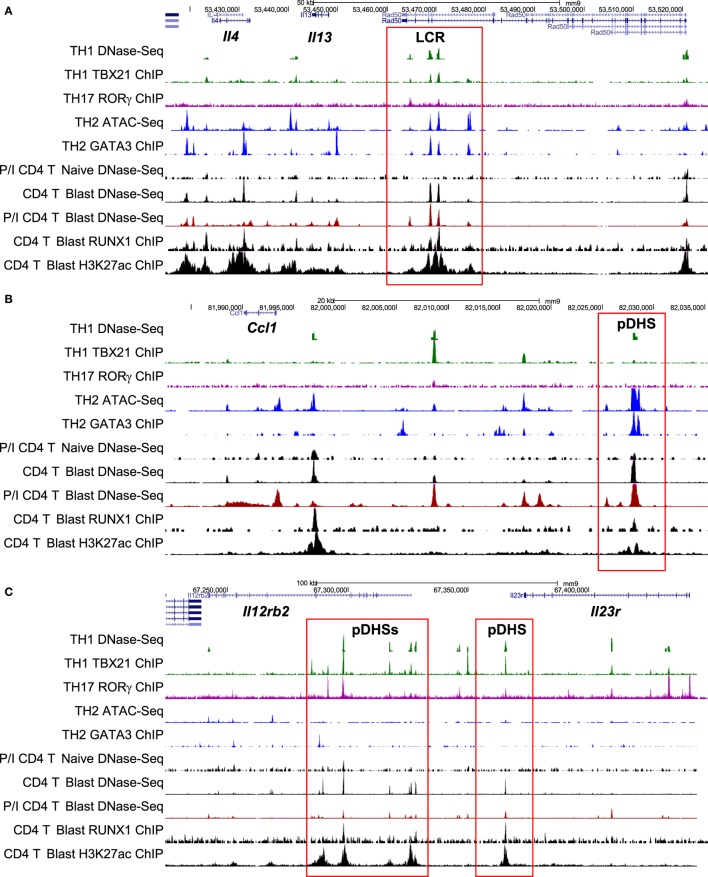
**Chromatin profiling of genes which are primed during blast cell transformation for subsequent binding of differentiation-inducing transcription factors**. Shown here are the previously defined profiles for the mouse type 2 helper (Th2) cytokine gene cluster **(A)**, the *Ccl1* locus **(B)**, and the *Il12rb2/Il23r* locus **(C)**, showing epigenetic profiles defined by Bevington et al. (10), plus other profiles for type 1 helper (Th1) DNase-Seq (89) and TBX21 ChIP-Seq (85), Th2 ATAC-Seq (90) and GATA3 ChIP-Seq (84), and Th17 RORγ ChIP-Seq (86). Note that ATAC-Seq is an alternative method for genome-wide profiling of DNase I hypersensitive sites (DHSs).

We find that many immune response genes, such as *CSF2* and *IL3*, reach their full potential for transcriptional activation during the initial blast cell transformation, showing little difference in inducible mRNA expression between undifferentiated T blast cells and Th1 or Th2 cells, or between CD4 and CD8 T blast cells (23). However, other genes, such as *IL4* and *IFNγ*, are primed during blast cell transformation but are only expressed at peak levels following further differentiation in the presence of polarizing cytokines, as depicted in Figure 1A. For the purpose of this review, we performed additional analyses of public data sets with a view to identifying other functions for pDHSs at later stages of T cell differentiation. We observed that subsets of the pDHSs that are formed during blast cell transformation are predicted to subsequently play additional roles in supporting the gene regulation networks that are established by newly induced master regulator TFs under polarizing cytokine gene conditions, as depicted in Figure 6A. Although pDHSs arise prior to helper T cell differentiation, some of these pDHSs, such as those seen in the Th2 cytokine gene locus, encompass GATA motifs (Figure 3D). For example, the *Rad50* +52 kb DHS in the Th2 LCR encompasses three GATA motifs (10), and many of the pDHSs in this locus recruit GATA3 after Th2 differentiation (Figure 7A).

To investigate the role of pDHSs in Th differentiation on a more global scale, we looked for the overlap between the 2,882 pDHSs that we defined in CD4 T blasts cells and the previously defined ChIP-Seq peaks taken from published studies of (i) GATA3 in Th2 cells, (ii) TBX21 in Th1 cells, and (iii) RORγ in Th17 cells (Figures 6B,C). Significantly, we found that 23% of the pDHSs were capable of recruiting TBX21, and 42% of the pDHSs were capable of recruiting GATA3 following differentiation.

To compare the relationship between pDHSs and the polarized mRNA expression patterns in differentiated cells (Figure 6A), we also compared the above ChIP-Seq and pDHS data with published mRNA data for the same cell types (Figure 6C). This allowed us to identify clear examples where elevated mRNA profiles were matched by recruitment of polarizing TFs to pDHSs. Hence, pDHSs recruit GATA3 to *Il4* and *Ccl1* in Th2 cells, TBX21 to *Gzmb* in Th1 cells, and RORγ to *IL23r* and *Il17ra* in Th17 cells. Interestingly, Th1 cells are closely related to Th17 cells, and we see a distinct overlap in the pDHS and ChIP-Seq profiles at genes such as *Ifgr1* and *Il12rb2* which are upregulated in both Th1 and Th17 cells (Figure 6C). Additional examples of specific pDHSs associated with locus polarization can be seen in Figure 7B where GATA3 is recruited to pDHSs within the *Ccl1* locus in Th2 cells, and in Figure 7C where TBX21 and/or RORγ are recruited to pDHSs within the *Il12rb2/Il23r* locus following Th1 or Th17 differentiation.

## The Requirement for Cytokine Signaling to Reinforce Immunological Memory

**Figure 8:**
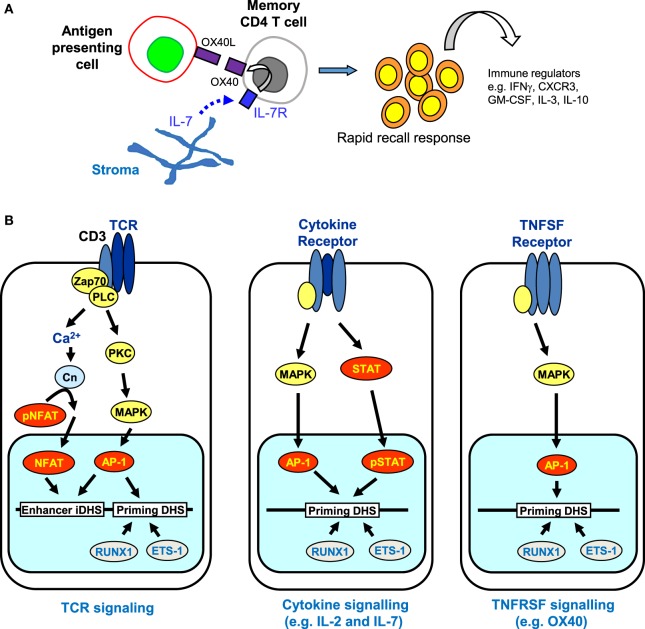
**The role of receptor signaling in immunological memory**. **(A)** Long-term immunological memory in CD4 T cells is dependent on cytokine and TNFSF networks involving interleukin 7 (IL-7) and OX40 that maintain the rapid recall response in CD4 memory T cells. Stroma-derived IL-7 upregulates expression of OX40, the receptor for OX40L, which is produced by antigen-presenting cells. **(B)** Multiple signaling pathways are predicted to reinforce epigenetic priming in memory T cells. T cell receptor (TCR) signaling activates both NFAT and AP-1 that cooperate to transiently induce DNase I hypersensitive sites (DHSs) at enhancers and trigger the establishment of primed DHSs. Once priming is established, cytokine and TNFSF signaling pathways can subsequently induce AP-1 and/or STAT family transcription factors (but not NFAT) which are predicted to reinforce the priming that was initially introduced *via* TCR signaling.

Despite the apparently stable nature of priming at pDHSs, it is well established that the maintenance of acquired immunity is itself highly dependent upon continual reinforcement from various cytokine and TNF receptor super family (TNFRSF) signaling networks (91–94). The requirement for gamma chain cytokines, particularly interleukin 7 (IL-7), for the survival of naïve and memory T cells is well documented (95, 96). Memory CD4 T cells were initially divided into two subsets based on their expression of molecules enabling recirculation through lymph nodes (a CCR7 and CD62L-dependent process) and their cytokine profile after restimulation (97, 98). The term “central memory” described those CD4 T cells that expressed CCR7 and so could traffic through lymph nodes, but produced mostly IL-2 in the absence of effector cytokines; “effector memory” defined those cells lacking expression of CCR7, but able to rapidly produce effector cytokines such as IFNγ upon restimulation (97, 98). Thus memory CD4 T cell populations appear to divide the labor of surveying the periphery for signs of re-infection, while also being positioned to re-expand T cell populations within secondary lymphoid tissue and support other adaptive immune cells. More recently, analysis of endogenous Ag-specific memory CD4 T cells generated in a systemic Th1 response has provided data consistent with this nomenclature. Tracking 2W1S-specific responses induced by attenuated *Listeria monocytogenes*, the CCR7−CXCR5− memory population rapidly produced IFNγ and IL-2, but only gave rise to more effector memory cells, while the CCR7+CXCR5+ cells generated mostly IL-2 but could generate both effector and follicular CD4 T cell subsets (99). There is evidence that Th1 effector memory cells are dependent on signaling through the TNF receptor super family protein OX40 (TNFRSF4) (100, 101). Furthermore, an OX40-deficient human has been described (91), who had no *in vitro* detectable recall responses, while nevertheless clearly being able to control most infections. Strikingly, despite being vaccinated three times with live attenuated BCG vaccine, no detectable *in vitro* or *in vivo* responses were detected, although the patient did not succumb to BCG disease. This phenotype of failing to mount rapid recall responses indicates a lack of, or impairment within the effector memory cell compartment. Mice in which OX40 is lacking show a similar defect in CD4 T cell recall responses (93, 102). Interestingly, Ag-experienced T cells rapidly respond to IL-7 *ex vivo* to upregulate expression of OX40, a process that does not occur in naïve CD4 T cells (93), suggesting that priming of the *OX40* locus is maintained in these cells. The accumulated evidence from OX40-deficient humans and mice would suggest that in, for example, an activated lymph node where Ag-presenting cells were induced to express OX40-ligand (TNFSF4), engagement of OX40 on IL-7-stimulated memory CD4 T cells would facilitate rapid responses to Ag re-exposure to produce effector cytokines (Figure 8A).

Taken together, these observations raise the additional prospect that various alternative signaling pathways, which are required for the survival of memory T cells, could be involved in maintaining the pDHSs in the absence of TCR activation (Figure 8B). Once pDHSs have been created in response to AP-1 *via* TCR signaling, these same sites may be reinforced by intermittent recruitment of AP-1 and/or STAT family proteins, or other inducible factors which are activated by IL-7 or TNFRSF signals (103–107) (Figure 8B). Indeed, motif analyses showed that STAT and AP-1 sites are enriched within the pDHSs (Figure 3D) and footprinting data suggests that some of the STAT sites are bound in the memory T cells (10). Furthermore, this view is supported by the *in vitro* experiments using the actively dividing T blast cells cultured with IL-2 which also have STAT and AP-1 sites footprinted in the pDHSs (10). Since these cells were constantly exposed to the gamma chain cytokine IL-2, which is closely related to IL-7, it seems that the activation of STAT5 and induction of AP-1 downstream of these signals may be a common mechanism to maintain the pDHSs in an open conformation in both the actively proliferating T blast cells and the quiescent memory T cells.

Another intriguing possibility is that STAT5 is acting as a molecular “bookmark” which is later replaced by STAT4, STAT6, or STAT3 when the cells are exposed to changes in signals and polarized to either Th1, Th2, or Th17 cells. An alternative, although not mutually exclusive, hypothesis is that STAT5 could be working as a transcriptional repressor. Studies in B cells have shown that STAT5 binding is associated with the prevention of immunoglobulin kappa light chain recombination *via* a block in E2A binding and also through recruitment of the histone methyltransferase EZH2 (108, 109). STAT5 could, therefore, be interacting with the pDHSs to suppress gene expression while maintaining a primed conformation for when the cells are re-activated.

## Concluding Statement

**Figure 9:**
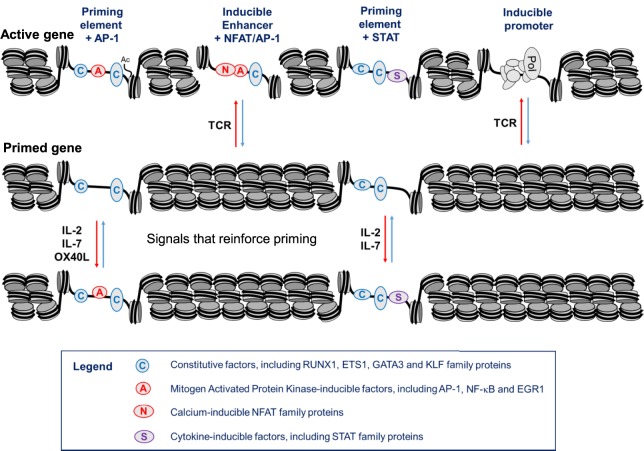
**Hypothetical model of a gene locus which is stably primed in memory T cells**. In this model, T cell receptor (TCR) signaling initially establishes priming at primed DHSs (pDHSs) which is regularly reinforced when T cells are exposed to cytokine and/or TNFSF signaling as depicted in Figure 8. Either AP-1 or STAT proteins can be recruited to pDHSs. The STAT motifs found in subset of pDHSs are capable of binding STAT proteins linked to multiple cytokine signaling pathways, including IL-2, interleukin 7 (IL-7), IL-6, and IL-12, as depicted in Figure 1A.

Taken together, these studies suggest a model whereby immunological memory is maintained by epigenetic reprogramming which is first established *via* TCR signaling, then maintained by growth factors such as IL-2 and IL-7 during a proliferative response in activated T cells, and finally maintained by a cytokine network linked to TNFRSF signaling molecules in quiescent memory T cells (Figure 9). The key to this model is that tight regulation observed at many immune response genes is mediated largely at the level of chromatin accessibility, and acquired immunity is likely to require regular reinforcement of the mechanisms that maintain accessibility. Importantly, the epigenetic priming that underpins acquired immunity in T cells is maintained by a specific class of regulatory elements, distinct from conventional transcriptional enhancers, that function primarily to maintain active chromatin rather than directly activating transcription.

## Public Data from the Gene Expression Omnibus Used in the Analyses Described Here

In addition to multiple data sets defined by Bevington et al., this article describes

Mouse Th1 mRNA GSM1182981, GSM1182982 (87).
Mouse Th17 mRNA GSM1182977, GSM1182978 (87).
Mouse Th2 mRNA GSM1194889 (88).
Mouse Th2 GATA3 ChIP-Seq GSM523226 (84).
Mouse Th1 TBX21 ChIP-Seq GSM998272 (85).
Mouse Th17 RORγ ChIP-Seq GSM1004856 (86).
Mouse spleen CTCF ChIP-Seq GSM918745 (74).
Mouse Th1 DNase-Seq GSM836117 (89).
Mouse Th2 ATAC-Seq GSM2056326 (90).

## Author Contributions

SB, PC, and PNC analyzed the data and SB, DW, PL, and PNC wrote the manuscript.

## Conflict of Interest Statement

The authors declare that the research was conducted in the absence of any commercial or financial relationships that could be construed as a potential conflict of interest.
